# Understanding the immunomodulatory mechanisms for natural products that overcome tumor chemoresistance to platinum-based cancer drugs and other treatments

**DOI:** 10.3389/fphar.2026.1689095

**Published:** 2026-04-30

**Authors:** Yaoxuan Zhu, Enting Lu, Fangmei Li, Yaqi Fan, Jingxin Zhang, Lei Deng, Xinyuan Li, Tianzhi Zhang, Huayang Li, Yuning Wang, Miao Lin, Mingming Xia, Haiqing Jia, Dongli Tian, Yan Yan, Jing-Yan Han, Bingzhen Shang, Yin Li, Yi Zhang

**Affiliations:** 1 Department of Integration of Chinese and Western Medicine, School of Basic Medical Sciences, Peking University, Beijing, China; 2 Department of Gynecology, The First Hospital of China Medical University, Shenyang, Liaoning, China; 3 School of Nursing, Beijing University of Chinese Medicine, Beijing, China; 4 Cancer Hospital of China Medical University, Liaoning Cancer Hospital & Institute, Shenyang, Liaoning, China; 5 Department of Clinical Trial Ward, Clinical Trial and Conversion Center, Shengjing Hospital of China Medical University, Shenyang, Liaoning, China

**Keywords:** chemosensitivity, immunomodulation, natural products, platinum resistance, tumor drug resistance

## Abstract

**Rationale:**

Cancer remains one of the most formidable challenges in mordern medicine. Chemoresistance, particularly resistance to platinum-based agents, presents a major challenge in effective treatments. In response to this issue, cancer immunotherapy has emerged as a treatment approach that boosts the body’s immune system and cellular defenses to eliminate cancer cells, complementing the established pillars of cancer treatment: surgery, radiotherapy, and chemotherapy. Recently, natural products have attracted significant interest for their cancer immunotherapeutic properties as well as their potential to help overcome chemoresistance. Yet the underlying mechanisms remain poorly understood.

**Objective and Findings:**

In this review, we examine the dual roles of immune cells in tumor proliferation and elimination and discuss the application of natural products in alleviating and overcoming tumor drug resistance, specifically focusing on immunomodulatory mechanisms. Current evidence shows that specific natural products can attenuate chemoresistance by modulating the functions of T cells, macrophages, NK cells, and dendritic cells. Notably, baicalin, naringin, ginsenosides, and atractylenolide I can alleviate platinum resistance via immunomodulation, enhanced apoptosis, and inhibition of oncogenic pathways (NF-κB, PI3K/Akt, STAT3). These compounds show synergistic therapeutic potential in colorectal, lung, and ovarian cancers.

**Conclusion:**

Immunomodulation represents a promising strategy in overcoming tumor drug resistance. The use natural products as immunomodulatory agents to enhance tumor chemosensitivity may constitute a novel therapeutic strategy, providing a foundation for the development of novel adjuvant cancer therapies.

## 1 Introduction

Platinum-based chemotherapies, such as cisplatin, carboplatin, and oxaliplatin, constitue one of the most effective first-line treatments for various malignancies, including ovarian, lung, and colorectal cancer. These agents exert their effects by penetrating tumor cells and inducing DNA damage, thereby triggering cell cycle arrest and ultimately promoting programmed cell death. However, the resistance of tumor cells to these agents (which is commonly termed as platinum resistance), particularly cisplatin resistance, poses a significant challenge in clinical oncology, undermining treatment efficacy and contributing to poor patient prognosis (Jayson et al., 2014). The development of chemoresistance in tumors is a multifactorial process determined by a complex landscape of both intrinsic tumor cell properties and the dynamic tumor microenvironment (TME) (Lainetti et al., 2020; Lee et al., 2014; Garg et al., 2021).

At the cellular level, platinum resistance is primarily driven by the drug uptake capacity of tumor cells as well as their molecular efflux systems. Notably, one prominent mechanism involves enhanced efflux of platinum drugs from tumor cells through the downregulation of copper transporter 1 (CTR1) and upregulation of ATP7A/B, multidrug resistance proteins (MDR), and other drug transporters. These changes reduce platinum accumulation within tumor cells, ultimately reducing the cytotoxic effect of this treatment (Wu et al., 2016). Platinum drugs, once internalized by tumor cells, induce DNA damage through the promotion of intra- and interstrand DNA crosslinks, which hinder DNA replication, disrupt mitosis and trigger cell apoptosis. Moreover, drug-resistant tumors can even enhance DNA repair pathways, including nucleotide excision repair (NER) and homologous recombination (HR) that remove platinum-induced DNA adducts. Overexpression of repair proteins, including ERCC1, has also been linked to poor responses to platinum therapy in various types of cancers (Zhang et al., 2022).

Epigenetic modifications to tumor cell DNA have been found to contribute to platinum resistance. Specifically, DNA methylation and histone modifications alter gene expression profiles and facilitate tumor survival under chemotherapeutic stress. These alterations, in combination with inactivating mutations or functional mutations to DNA repair-related genes, altogether contribute to the ability for tumor cells to evade the cytotoxic effects of platinum-based drugs (Ganesan et al., 2021). Additionally, tumor cells can also adapt by modulating cell death pathways, particularly through suppression of apoptotic signaling and activation of autophagy, which enables cells to persist despite substantial DNA damage that triggers such cell death responses. Together, such mechanisms enable tumor cells to resist chemotherapy, promoting survival and recurrence despite prolonged treatment (Ashrafizadeh et al., 2020).

In addition to the intrinsic properties of tumor cells to confer platinum resistance, the TME also plays pivotal roles. The TME is a complex ecosystem comprising immune cells, fibroblasts, endothelial cells, and extracellular matrix components. Tumor-associated macrophages (TAMs), cancer-associated fibroblasts (CAFs), and myeloid-derived suppressor cells (MDSCs) contribute to platinum resistance by secreting growth factors, cytokines, and chemokines that support tumor survival, angiogenesis, and immune evasion (Babaei et al., 2021; Wu et al., 2016). These cells create protective niches that reduce drug penetration and contribute to immune suppression, further complicating treatment strategies (Liu et al., 2022a).

Cancer immunotherapy has emerged as the fourth pillar of cancer treatment alongside surgery, chemotherapy, and radiotherapy, offering a promising strategy for overcoming platinum resistance (Dong et al., 2022). Immune checkpoint inhibitors (ICIs) such as anti-PD-1/PD-L1 antibodies, and T-cell therapies such as CAR-T cells have all been useful as cancer immunotherapy treatments for several types of cancer, but their effectiveness is often limited by TME-mediated immune evasion (Dall'Olio et al., 2022; Propper and Balkwill, 2022). Moreover, the infiltration of immunosuppressive cells including regulatory T cells (Tregs) and MDSCs represent a major mechanism of immune evasion which inhibits the activity of anti-tumor immune cells such as cytotoxic T lymphocytes (CTLs). Also, tumor cells can activate immune checkpoints (e.g., PD-1/PD-L1) to suppress T cell activity, contributing to immune resistance. Our further understanding of cancer immunotherapy will improve our capacity to strategically modulate the immune system to treat cancer.

In recent years, natural products have emerged as promising candidates for overcoming platinum resistance through their actions to modulate the TME and enhance immune responses in order to eliminate tumor cells. Indeed, natural compounds such as baicalin, ginsenosides, and curcumin have been found to alleviate cisplatin resistance by regulating immune cell functions and reprogramming the TME (Ganesan et al., 2021). Specifically, natural products can enhance the cytotoxicity of CD8^+^ T cells, restore antigen presentation by dendritic cells (DCs), and modulate macrophage polarization, thereby improving tumor immunosurveillance and counteracting the immunosuppressive effects of the TME (Wan et al., 2022; Yeh et al., 2017). Furthermore, natural products such as resveratrol, baicalein, and ginsenosides have demonstrated synergistic effects when combined with platinum-based therapies to treat cancer, enhancing treatment efficacy while maintaining relatively favorable toxicity profiles compared to traditional chemotherapies (Yeh et al., 2017; Gandhi et al., 2018). These findings suggest that natural products may serve as valuable immunomodulatory agents that enhance the efficacy of platinum-based chemotherapy, offering a synergistic interaction that concurrently targets tumor cells and modulates the immune system.

Overcoming platinum resistance requires a multifaceted approach that targets both the intrinsic mechanisms of tumor cells and the immunosuppressive TME. By leveraging natural products as immunomodulators, it is possible to enhance chemosensitivity, reverse resistance, and ultimately improve treatment outcomes in patients with platinum-resistant cancer. In the following sections, we synthesize current knowledge on the role of the immune system in cancer resistance, exploring the effects and mechanisms through which natural products act as immunomodulators to reverse drug resistance in both preclinical and clinical contexts.

## 2 The role of immune cells in tumor drug resistance

Chemotherapy is a critical treatment pillar for comprehensive cancer treatment of patients. However, the efficacy of chemotherapy is limited by the development of drug resistance in tumor cells, contributing to cancer recurrence and reduced patient survival (Vasan et al., 2019). Platinum resistance is a complex mechanism that involves adaptive changes in tumor cells and the regulation of the TME (Srivastava et al., 2019).

Immune cells interact with tumor cells in the TME, and may mediate drug resistance through multiple mechanisms, including preventing the immune clearance of tumor cells, hindering drug absorption, and stimulating paracrine growth factors to signal cancer cell growth (Sharma et al., 2017). The TME is a complex ecosystem in which immune cells play a significant role in tumor occurrence, development, and the body’s response to chemotherapy drugs (Arora and Pal, 2021). In recent years, the dynamic regulatory role of immune cells in the TME has been the focus of significant scientific research. The TME has been recognized as a factor for promoting drug resistance through cellular and molecular mechanisms in this niche including cytokine secretion, regulation of cell metabolism, and remodeling of the immunosuppressive microenvironment (Binnewies et al., 2018).

In this section, we explore how various immune cells participate in the development of drug resistance during tumor progression, aiming to provide theoretical basis and new treatment ideas for overcoming tumor drug resistance and identifying pathways for improving the overall efficacy of cancer treatment.

### 2.1 Types and functions of immune cells

Immune cells comprise at least 14 different cell types, categorized as innate and adaptive immune cells. Innate immune cells include macrophages, neutrophils, dendritic cells, eosinophils, mast cells, and natural killer cells, while adaptive immune cells are composed of T and B lymphocytes (Mao et al., 2021). Among central players in cellular immunity are T cells which can be further classified into helper (Th) and cytotoxic (CTL) T cells based on their functions (Tarannum et al., 2025). Th cells assist the activation of other immune cells, while CTL directly kill target cells (Sun and Dong, 2025). B lymphocytes predominantly participate in humoral immunity and produce antibodies. NK cells can recognize and kill target cells without prior sensitization, and play an important role in anti-tumor immunity (Chen and Mellman, 2017). Macrophages serve multiple functions, including phagocytosis, antigen presentation, and cytokine secretion. They can be classified into classically activated M1-type macrophages and alternatively activated M2-type macrophages based on their phenotypes and functions (Summer et al., 2025). M1-type macrophages serve anti-tumor activities, while M2-type macrophages have been reported to promote tumor growth and metastasis (Liu et al., 2022c). Dendritic cells (DCs) are highly potent antigen-presenting cells which can activate naive T cells, initiating adaptive immune responses (Zhang et al., 2024a).

### 2.2 Immune cell types of the TME and their mechanisms for tumor drug resistance

Tumor stem cells are endowed with the capacity for self-renewal and multi-directional differentiation, and their continuous proliferation during cancer drug treatment implies their lack of sensitivity to chemotherapy drugs (Walcher et al., 2020). There are two forms of resistance to tumor drugs, namely, primary resistance and acquired resistance. Primary resistance refers to the insensitivity of tumor cells to chemotherapy drugs from initial treatment, while acquired resistance refers to drug resistance that gradually develops in tumor cells during treatment. The mechanisms of tumor drug resistance are complex and diverse. For example, in solid tumors, the permeability of the tumor mass and the cellular overexpression of drug efflux pumps by tumor cells influence how chemotherapy drugs permeate into cells and accumulate intracellularly at effective concentrations or are pumped out, respectively (Yin et al., 2021). Abnormalities in apoptotic signaling pathways, observed as high levels of autophagy in tumor cells, are associated with resistance to systemic treatment. This clinical observation contributes to the notion that autophagy may promote the survival of stressed or damaged cells by recycling cellular breakdown products (Miller and Thorburn, 2021).

Tumor cells are known to secrete immunosuppressive factors to inhibit immune cell activation and function. On the other hand, immune cells can also affect tumor drug resistance by directly killing tumor cells or by influencing the TME. For this review, we focus on describing the mechanisms through which tumor cells evade immune surveillance in the TME, leading to impaired immune cell function and tumor cell elimination within the body. TME contributes to platinum resistance by creating protective niches and modifying cancer cell behavior. Within the TME, cell mechanistic signaling behaviors in tumor cells, such as epithelial-to-mesenchymal transition (EMT) and stemness, have been described as strategies to evade drug-induced death. Key components of the TME, including TAMs and CAFs, secrete factors that enhance cell survival, autophagy, and drug efflux. These interactions ultimately reduce the efficacy of platinum-based therapies, leading to treatment failure.

#### 2.2.1 T lymphocytes

##### 2.2.1.1 Th cells

Th cells are predominantly classified into Th1 cells and Th2 cells. Th1 cells promote cellular immune responses and secrete IFN-γ, which upregulates, major histocompatibility complex (MHC) class I molecules on the surface of tumor cells, thus enhancing the recognition and killing ability of CTLs against tumor cells (Xuan et al., 2022). Th2 cells exhibit immunosuppressive effects through inhibiting the maturation and function of DCs and reduce their antigen-presenting ability, thereby weakening the immune response of T cells against tumor cells. In addition, Th2 secrete cytokines that promote the polarization of tumor-associated macrophages to M2 type, further inhibiting the anti-tumor immune response and promoting tumor drug resistance (Li et al., 2022a). Th17 cells are a recently discovered subset of Th cells that mainly secrete cytokines such as IL-17. IL-17 can promote tumor angiogenesis, inflammatory responses, and the proliferation and migration of tumor cells. In ovarian cancer, IL-17 can upregulate the expression of multidrug resistance protein 1 (MRP1) in tumor cells, thereby enhancing drug efflux capacity and reducing intracellular cisplatin accumulation, ultimately contributing to the tumor chemoresistance (Zhang et al., 2020).

##### 2.2.1.2 Cytotoxic T lymphocytes (CTLs)

CTLs are one of the main effector cells that serve anti-tumor immunity roles. They are capable of specifically recognizing and killing tumor cells that express tumor antigens. Drug-resistant tumor cells can evade CTL recognition and killing by downregulating the expression of MHC class I molecules (Hu et al., 2024). Immunosuppressive factors such as TGF-β and IL-10 inhibit the expression of perforin and granzyme B in CTL, reducing their cytotoxic activity.

CTLs are essential effectors in the immune response against cancer. These cells specifically target and eliminate tumor cells expressing tumor-associated antigens in association with MHC class I molecules on their cell surface. The interaction between the T cell receptor on CTLs and the tumor antigens presented by MHC class I triggers a cytotoxic response, which leads to tumor cell apoptosis via perforin and granzyme B secretion. However, drug-resistant tumor cells can evade CTL-mediated killing through several immune escape mechanisms, which significantly contribute to the development of chemoresistance, particularly to platinum-based therapies. One key mechanism for immune escape is the downregulation of MHC class I expression, which prevents CTLs from recognizing and targeting tumor cells. MHC I downregulation in drug-resistant tumors is driven by various factors including genetic mutations, epigenetic alterations, and TME-induced modifications. These modifications inhibit antigen presentation and reduce the immunogenicity of tumor cells, rendering them less susceptible to CTL-mediated cytotoxicity. In addition, tumors can influence the levels of immunosuppressive cytokines including TGF-β and IL-10, which act on CTLs to downregulate perforin and granzyme B, thus allowing tumor cells to evade immune surveillance and resist chemotherapy (Xiao et al., 2019). Another mechanism of immune evasion is the activation of immune checkpoints, particularly the PD-1/PD-L1 axis. Tumor cells overexpressing PD-L1 bind to the PD-1 receptor on CTLs, which leads to T cell exhaustion and suppression of their cytotoxic function. This mechanism plays a pivotal role in the resistance of tumors to both immune and chemotherapeutic therapies. In this context, immune checkpoint inhibitors such as anti-PD-1 and anti-PD-L1 antibodies have demonstrated strong potential for reactivating CTLs and overcoming immune resistance across multiple cancer types (Liu et al., 2021).

Recent studies have shown that natural products enhance CTL activity and reverse immune suppression in the TME (Zhang et al., 2022). For example, certain flavonoids and alkaloids have been found to restore MHC class I expression, enhance cytokine production, and modulate immune checkpoints, thereby improving CTL-mediated tumor cell killing (Liu et al., 2022b). Such findings suggest that natural products may represent novel immunomodulatory therapies to treat cancer and overcome platinum chemoresistance.

##### 2.2.1.3 Other T lymphocytes

CD8^+^ T cells represent the core effector cells for anti-tumor immunity, yet their functions are often suppressed within the TME. Regulatory T cells (Tregs) inhibit the activities of CD8^+^ T cells by secreting IL-35 and TGF-β, while promoting the self-renewal of cancer stem cells (CSCs), which are naturally resistant to cisplatin (Wu et al., 2023). Moreover, PD-1/PD-L1 signaling is abnormally activated in ovarian cancer, leading to T cell exhaustion and platinum resistance (Horikawa et al., 2017). Evidence from tumor tissue samples suggests that high expression of PD-L1 is associated with shortened progression-free survival in ovarian cancer patients after platinum-based drug treatment (Martinez-Usatorre et al., 2021).

#### 2.2.2 Tumor-associated macrophages (TAMs)

TAMs are the most abundant immune cell population within the TME. M1-type macrophages perform anti-tumor activity and can secrete large quantities of pro-inflammatory cytokines, including IL-1, IL-6, TNF-α, to activate T cells and NK cells and enhance the anti-tumor immune response (Wu et al., 2024). However, TAMs in the TME are usually polarized to M2-type macrophages, which secrete factors such as IL-10 and TGF-β. These factors promote tumor cell proliferation, metastasis, and angiogenesis, and inhibit the anti-tumor immune response (Mantovani et al., 2022). Studies have shown that TAMs promote cisplatin resistance in ovarian cancer cells by activating the STAT3 pathway, such that STAT3 in turn upregulates the expression of anti-apoptotic protein Bcl-2 and enhances DNA damage repair functions in cells (Luo et al., 2021). Additionally, TAMs secrete CCL18, which can induce EMT in tumor cells, further promoting tumor cell invasion and drug resistance (Ma et al., 2021). Such functions for TAMs in TME have also been reported in ovarian cancer, with a study reporting high proportions of M2-type TAMs in the ascites of ovarian cancer patients are significantly associated with cisplatin resistance. Interestingly, removal of TAMs has been reported to restore chemotherapy sensitivity (Zheng et al., 2023). Moreover, M2-type macrophages have also been reported to utilize and metabolize arginine in the TME through their expression of enzymes including arginase 1 (Arg1), inhibiting T cell proliferation and function and promoting platinum drug resistance in ovarian cancer (Li et al., 2022a).

#### 2.2.3 Natural killer (NK) cells

NK cells are an important component of the innate immune system. They possess non-specific cytotoxicity. NK cells can directly kill tumor cells by releasing perforin and granzyme B. They can also regulate the function of immune cells in the TME by secreting cytokines such as IFN-γ and TNF-α. Tumor cells resistant to platinum-based drugs may express ligands such as human leukocyte antigen-G (HLA-G) that inhibit the activities of NK cells and weaken their ability to kill tumor cells (Zheng et al., 2021). The function of NK cells is inhibited by TGF-β and adenosine in the TME. HLA-G is highly expressed in platinum-resistant ovarian cancer cells, which can escape immune surveillance by binding to KIR2DL4 on the surface of NK cells (Zheng et al., 2022).

#### 2.2.4 Dendritic cells (DCs)

DCs serve as a link between innate and adaptive immunity. Mature DCs can take up, process, and present tumor antigens. They activate naive T cells and initiate adaptive immune responses. However, in the TME, the functions of DCs have been found to be suppressed, leading to reductions in both their antigen presentation and T activation functions. For example, tumor cells can secrete immunosuppressive factors such as TGF-β and IL-10 to inhibit the maturation and function of DCs. Moreover, high concentrations of metabolic products such as lactic acid in the TME can also affect the function of DCs. Studies have shown that the use of DC vaccines to enhance the function of DCs can increase the sensitivity of ovarian cancer patients to cisplatin and thus enhance the anti-tumor immune response (Chen et al., 2024a).

#### 2.2.5 Myeloid-derived suppressor cells (MDSCs)

**FIGURE 1:**
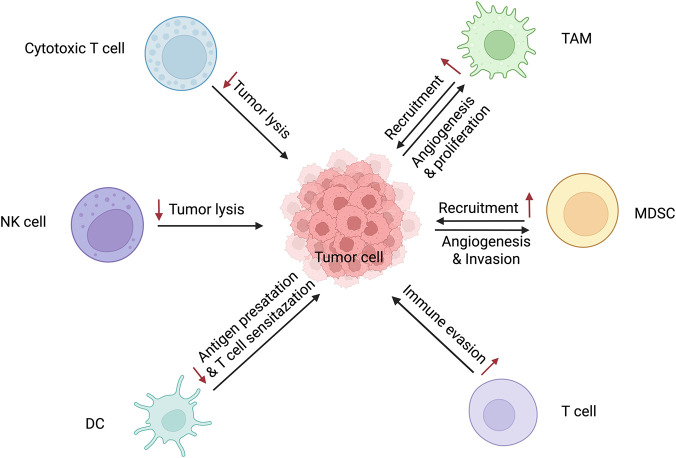
Main Immune Cell Types in the TME and Their Mechanisms Related to Tumor Drug Resistance (Image created using https://BioRender.com).

MDSCs are heterogeneous populations of myeloid progenitors and immature bone marrow cells. They act as key immunosuppressive components of the TME, migrating from bone marrow to tumors to promote immunosuppressive niches that facilitate tumor survival, angiogenesis, and metastasis (Kumar et al., 2016; Qu et al., 2016). Allopurinol and cediranib can reduce the infiltration of MDSCs through targeting arginase-1 and VEGFR, respectively, thus restoring the function of CD8^+^ T cells. Signal transducers and activators of STAT3 inhibitor, including Napabucasin, have also been shown to alleviate cisplatin resistance in preclinical models (Dong et al., 2023). The major immune cell types in the tumor microenvironment (TME) and their mechanisms associated with tumor drug resistance are illustrated in the Figure 1.

## 3 Therapeutic roles for natural products that alleviate and overcome tumor resistance

Natural products derived from plants and marine organisms have been widely adopted for use in conventional medicine (Liu et al., 2023). The majority of biologically active natural products that are beneficial for use to support patient survival in cancer are secondary metabolites and found predominantly to function by mediating the innate immune response to cancer. Approximately 47% of anti-tumor agents used in clinical practice have been sourced from natural products or their derivatives (Cadoná et al., 2022). Remarkably diverse in structure, function, and mechanism of biosynthesis, natural compounds that exhibit anticancer characteristics are categorized into terpenoids and steroids, alkaloids, fatty acid derivatives and polyketides, or phenylpropanoids (Cao et al., 2025). Alkaloid-based drugs have potent anticancer effects accompanied by relatively high toxicity, while terpenoids and flavonoids display moderate anticancer activity and are more tolerable (Zhou et al., 2025b). The diverse structural and biological features of such natural products have also informed the discovery and further development of drugs for cancer, including paclitaxel (derived from *Taxus brevifolia*), elemene emulsion, camptothecin (irinotecan), and compound Kushen injection (Li et al., 2024; Yuan et al., 2016).

In this section, natural products are systematically classified based on their chemical structures and biological properties, both of which critically influence their anticancer effects. These categories include alkaloids, flavonoids, terpenoids, phenolic acids, and other related compounds. A detailed overview of each category of natural product and their relevance in cancer treatment are presented below.

### 3.1 Alkaloids

Natural products categorized as alkaloids represent a group of structurally diverse organic compounds that mostly contain basic nitrogen atoms. Alkaloids are widely present in plant tissues (Tilaoui et al., 2021). Many anticancer agents are derived from alkaloids and their derivatives (Li et al., 2020; Laskar et al., 2019).

#### 3.1.1 Berberine

Berberine (BBR) is a naturally occurring bioactive alkaloid extracted from the herb *Coptis chinensis*. The therapeutic efficacy of BBR has been reported in several studies in cases of colon, breast, pancreatic, liver, oral, bone, cutaneous, prostate, intestine, and thyroid cancers (Rauf et al., 2021). BBR potentiates the sensitivity of gastric cancer cells to cisplatin resistance through modulation of MRP1 expression, enhancement of cancer cell apoptosis, and repression of PI3K/AKT/mTOR signaling (Kou et al., 2020). Beyond effects to overcome or alleviate cisplatin resistance, acquired resistance to epidermal growth factor receptor-tyrosine kinase inhibitors (TKIs, e.g., icotinib) also represents a significant clinical challenge in non-small cell lung cancer (NSCLC) treatment. BBR/icotinib combination synergistically induces autophagic cell death and apoptosis in EGFR-TKI-resistant NSCLC via EGFR autophagic degradation and ROS-mediated EMT inhibition, both *in vitro* and *in vivo* (Chen et al., 2021b).

#### 3.1.2 Camptothecin

Camptothecin (CPT) is a pentacyclic alkaloid derived from the stem of *Camptotheca acuminata*. CPT is a potent anti-cancer agent that targets topoisomerase I (TOP1), while its poor pharmacokinetic properties, high cellular toxicity, and high expectation of drug resistance significantly limit its broader clinical use (Chen et al., 2024b).

Aloin is a natural anthraquinone glycoside extracted from the plant *Aloe vera*. The combination of CPT-11 and aloin can activate miRNA-133b which, in turn, suppresses PI3K/AKT/mTOR and MEK/ERK signaling pathways via IGF1R downregulation, thus inhibiting colorectal cancer (CRC) progression (Jassi et al., 2023).

#### 3.1.3 Matrine

Matrine extracted from *Sophora flavescens* has various biological activities. In various types of cancers, matrine exhibits significant antiproliferative and proapoptotic properties (Pu et al., 2018). Under hypoxic conditions, p53 can suppress hypoxia-induced HIF-1α production by enhancing MDM2-mediated ubiquitination and proteasomal destruction, thus increasing the efficacy of cisplatin in treating liver cancer (Dai et al., 2021).

TME has been suggested to be a crucial factor in CRC progression, and CAFs are one of the key types of stromal cells (de Visser and Joyce, 2023). In CRC, matrine is shown to reduce the secretion of exosomal circSLC7A6 from CAFs, and inhibit tumorigenesis by regulating CXCR5 expression (Chen et al., 2021a).

#### 3.1.4 Other alkaloids

T-cell exhaustion is a major challenge in cancer immunotherapy, observed in part as prolonged activation of immune checkpoint inhibitor (ICI) pathways, including PD-1/PD-L1 and CTLA-4 signaling pathways (Qin et al., 2024). Addressing this challenge, Picrasidine S, originating from *Picrasma quassioides*, is a novel cancer vaccine adjuvant that can significantly enhance both humoral and cellular immune responses. At the molecular level, picrasidine S amplifies CD8^+^ T cell-mediated antitumor immune response by activating the cGAS-IFN-I pathway (Ding et al., 2024). Moreover, ligustrazine (LSZ), an amide alkaloid derived from *Ligusticum chuansiong Hort*., exhibits antiproliferative effects on multiple cancer cell types by elevating ROS levels. LSZ derivatives also showed their potential to reverse cisplatin resistance in combination therapy by potently inhibit the proliferation of drug-resistant hepatocellular carcinoma (Qian et al., 2021). Another alkaloid, piperlongumine (PL), is a natural alkaloid derived from *Piper longum* (long pepper), exhibits *in vitro* antitumor and antiproliferative activities. PL enhances natural killer (NK) cell-mediated cytolysis of tumor cells without impairing NK cell function (Afolabi et al., 2021).

### 3.2 Flavonoids

Flavonoids are heterocyclic natural organic compounds containing nitrogen, including flavones, flavonols, and chalcones (Leonte et al., 2023). Flavonoids are highly abundant in green plants, including several traditional Chinese herbs, and have been shown to exhibit beneficial effects in facilitating immunoregulation and overcoming tumor drug resistance (Rengasamy et al., 2019; Wen et al., 2020).

#### 3.2.1 Baicalin and baicalein

Baicalin and baicalein are naturally occurring flavonoids, and the primary bioactive constituents found in the dried rhizome of *Scutellaria baicalensis*. Baicalein is the aglycone of baicalin. Studies have shown that these flavonoids overcome function in the body to overcome multidrug resistance by modulating the tumor immunosuppressive microenvironment in various types of cancers (Cai et al., 2021). Baicalin can reduce CRC cell viability and inhibit proliferation by triggering mitochondrion-mediated apoptosis. Baicalin was also shown to suppress migration and invasion of CRC cells by suppressing the TLR4/NF-κB signaling pathway (Song et al., 2022). In preclinical models of colorectal cancer, baicalin has been shown to enhance anti-tumor immunity by increasing immune cell activity and cytokine production, partly through modulation of the TLR4/NF-κB signaling pathway, thereby inhibiting tumor growth and progression (Wang et al., 2024). Furthermore, baicalin enhanced the infiltration of CD4^+^ and CD8^+^ T cells into the TME, resulting in a more robust anti-tumor immune response. This effect appears to be mediated by the upregulation of CXCL9 via the HIF-1α/lactate pathway, which facilitates T cell recruitment and activation (Yang et al., 2025). Additionally, Baicalin treatment significantly reduced the proportion of MDSCs, which are known to inhibit T cell activity and promote tumor progression. Baicalin also downregulated the expression of PD-L1 on tumor cells, a key immune checkpoint molecule that dampens T cell-mediated immune responses. As a result, the modulation of these immune components by Baicalin led to enhanced T cell cytotoxicity and suppression of immune escape mechanisms, ultimately contributing to its anti-tumor efficacy. These findings suggest that Baicalin may function as an immunomodulatory agent, improving the therapeutic response in CRC and offering potential for clinical application (Song et al., 2022). As for baicalein, Jin et al. demonstrated that baicalein exposure induces cuproptosis, a copper ion-mediated form of cell death through the activation of Akt, thus potentially enhance the sensitivity of cervical cancer cells to cisplatin (Jin et al., 2024).

#### 3.2.2 Resveratrol

Resveratrol (RES) is a polyphenol that is present in grapes. Sun et al. demonstrated that RES attenuates M2 macrophage polarization in human monocyte-derived macrophages (HMDMs) induced by lung cancer cell-conditioned medium, while concurrently inhibiting the proliferation of lung cancer cells in macrophage co-culture systems (Sun et al., 2017). RES also exhibits anti-tumor effects in ovarian cancer cells by inducing immunogenic cell death (ICD), and mitigates osimertinib resistance by targeting BCL2L11 to promote apoptosis in lung cancer (Yu et al., 2025).

#### 3.2.3 Quercetin

Quercetin is a flavonoid widely present in fruits and vegetables, and as well as in tea. Studies in the last decade have shown that clinical use of quercetin has protective effects against various diseases, including several forms of cancer (Azeem et al., 2023). Preclinical evidence indicates that quercetin enhances the effectiveness of cisplatin in cervical cancer cells (e.g., HeLa/SiHa cells) by downregulating *METTL3*, *MMP2*, *EZR* (encodes ezrin), and *PGP* (encodes P-glycoprotein, P-gp) (Xu et al., 2021b; Shao et al., 2024). Quercetin is also thought to alleviate 5-FU/oxaliplatin resistance in CRC cells through METTL3 inhibition, underscoring METTL3 as a critical target for quercetin-mediated chemosensitization (Zeng et al., 2020; Xu et al., 2021b; Shao et al., 2024).

#### 3.2.4 Curcumin

Curcumin is a natural phenol derived from the medicinal spice turmeric (*Curcuma longa*). Curcumin enhances the antitumor efficacy of irinotecan in CRC cell lines LoVo and HT-29 by inducing apoptosis and cell cycle arrest (Xu and Shen, 2024). This effect is mediated through elevated cellular reactive oxygen species (ROS) and activation of endoplasmic reticulum (ER) stress (Xu and Shen, 2024). Furthermore, curcumin reduced the abilities of migration and invasion of BC cells as well as lung metastasis of breast cancer in a nude mouse model involving TEAD4 induction (Li et al., 2025b).

#### 3.2.5 Epigallocatechin gallate

Studies have shown that epigallocatechin gallate (EGCG), the most abundant polyphenol in green tea, exhibits preclinical anticancer activity against multiple cancers in both *in vitro* and *in vivo* models (Oliveira et al., 2016). EGCG exerts dose-dependent inhibitory effects on cisplatin-resistant oral cancer CAR cells by downregulating *MDR1* expression, thus reducing phosphor-AKT (Ser473)/STAT3 and Bcl-2 levels (Yuan et al., 2017). EGCG also functions as an inhibitor of DNA methyltransferase (DNMT). In A549 non-small cell lung cancer cells, EGCG exposure causes loss of DNA methylation at *GAS1*, *TIMP4*, *ICAM1*, and *WISP2* genes, thereby enhancing cisplatin sensitivity by modulating DNA methylation-dependent cellular responses to chemotherapy (Singh et al., 2011; Li et al., 2022b).

#### 3.2.6 Other flavonoids

Amentoflavone (AF) enhances cisplatin-induced apoptosis of oral squamous cell carcinoma (OSCC) cells through a mechanism involving NF-κB inactivation and both caspase-dependent and -independent intrinsic pathways, altogether suggesting its potential therapeutic benefit as a cancer treatment (Chen C.-H. et al., 2020). Scutellarin enhances cisplatin-induced tumor cell apoptosis and autophagy in non-small-cell lung cancer (NSCLC) through a mechanism involving ERK-p53 activation, and inhibition of the c-met-AKT signaling pathways (Sun et al., 2018). Scutellarin can also synergistically enhance the anticancer effects of cisplatin on ovarian cancer cells by improving cisplatin-DNA binding affinity (Xie et al., 2019). Naringin is a water-soluble flavonoid that is abundant in citrus fruits, such as grapefruit and oranges. In addition to its well-established antioxidant and anti-inflammatory properties, Naringin has been shown to exert immunomodulatory effects by regulating inflammatory signaling pathways and cytokine production. For example, Naringin has been reported to suppress pro-inflammatory cytokines such as IL-6 and TNF-α and inhibit activation of the NF-κB signaling cascade, thereby reducing inflammation and potentially limiting tumor-associated immunosuppression, as demonstrated studies using *in vitro* and *in vivo* models (Yang et al., 2021). Moreover, studies suggest that Naringin and its aglycone metabolite naringenin can modulate immune cell functions, including macrophage activity and inflammatory mediator production (Kawaguchi et al., 2022). These mechanisms may contribute to improved anti-tumor immunity and enhanced responsiveness to chemotherapeutic agents through dampening of NF-κB–mediated immune suppression and microenvironmental inflammation (Stabrauskiene et al., 2022).

### 3.3 Terpenoids

Terpenoids are a structurally diverse class of natural products derived from five-carbon isoprene units and represent one of the largest groups of plant secondary metabolites of great structural and functional diversity. Beyond their prominent anticancer activities, such as induction of apoptosis, suppression of angiogenesis, and inhibition of tumor growth, terpenoids also exert significant immunomodulatory and anti-inflammatory effects, influencing cytokine production and immune cell function, which are postulated to improve antitumor immunity and influence the immunosuppressive TME, such as through modulating pro-inflammatory signaling and immune cell activation, in various cancer models (Kamran et al., 2022).

#### 3.3.1 Ginsenosides

Ginsenosides, the primary bioactive components of ginseng, are a class of sapogenins that exhibit various pharmacological effects, including inhibition of tumor cell growth, invasion, and microvascular formation, and induction of tumor cell apoptosis (Qu et al., 2023).

Multiple ginsenosides have been established as clinical treatments for the prevention and treatment of colorectal (Zhao et al., 2022), lung (Peng et al., 2020), and breast cancers (Fan et al., 2022). It was reported that Ginsenoside Rg3 (abbreviated as Rg3 in this review) could suppress the expression of PD-L1 in A549/DDP cells and restore T cell functions in in the context of chemoresistant NSCLC (Jiang et al., 2017). Rg3 also induces ICD, enhancing interferon-γ (IFN-γ) secretion to inhibit tumor growth (Son et al., 2016). Ginsenoside Rh2 represses EGFR/PI3K-AKT signaling, autophagy, and cisplatin-induced PD-L1 expression in lung adenocarcinoma cell models A549 and H1299 (Chen et al., 2020b). Ginsenoside Rg6 mitigates cisplatin resistance in EOC cells by suppressing fucosylation, inducing autophagy via GRB2-ERK1/2-mTOR inhibition, and enhancing cisplatin uptake through CTR1 upregulation (Xue et al., 2025). Ginsenoside Compound K (CK) exhibits the potential to overcome oxaliplatin (L-OHP) resistance in gastric cancer cells by inhibiting the PI3K/Akt pathway to reduce P-gp expression, and reverse EMT in *in vitro* and xenograft models, suggesting the potential for ginsenoside CK as an adjuvant to enhance L-OHP efficacy in chemoresistant patients (Zhang et al., 2025a).

#### 3.3.2 Cycloastragenol

Cycloastragenol (CAG), a metabolite of astragaloside IV derived from *Astragalus membranaceus.* In MC28 and CT26 mouse transplanted tumor models, CAG is shown to inhibit tumor growth *in vivo* by binding cathepsin B to block lysosomal degradation of MHC ClassI molecules and promotion of tumor cell-surface antigens, altogether enhancing the tumor killing function of CD8^+^ T cells (Deng et al., 2022). Additionally, its use in combination with PD-1 antibody has a more potent effect in xenograft mice models and CRC organoids (Deng et al., 2022).

#### 3.3.3 Triptolide and demethylzeylasteral

Triptolide (TPL) is the most potent bioactive molecule derived from the traditional Chinese medicine *Tripterygium wilfordii Hook. F* extract. TPL enhances chemosensitivity to cisplatin treatment in cisplatin-resistant human epithelial ovarian cancer SKOV3/DDP cells by inhibiting the phosphorylation of PI3K-Akt signaling and increasing Caspase-3-mediated apoptosis (Huang et al., 2019). Demethylzeylasteral (Dem) is another bioactive triterpene compound extracted from *T. wilfordii Hook F.* Dem exposure is shown to downregulate PD-L1 expression in CRC cells, thereby enhancing T cell-mediated tumor cytotoxicity and limiting tumor progression (Zhang et al., 2024b; Chen et al., 2022).

#### 3.3.4 Oridonin

Oridonin, a diterpenoid lactone isolated from *Isodon rubescens*, has demonstrated therapeutic potential in modulating tumor cell survival and apoptosis. It enhances NK cell-mediated cytotoxicity against lung cancer cells by modulating degranulation markers, cytokine secretion, NK cell surface receptor expression, and tumor cell ligand expression (Hwang and Chang, 2023). Additionally, Oridonin improves chemotherapy efficacy by modulating immune responses, such as upregulating PTEN and inhibiting PI3K/Akt signaling, which further enhances NK cell activity and reduces tumor resistance to chemotherapy agents such as paclitaxel and cisplatin (Chauhan et al., 2025).

#### 3.3.5 Tanshinone

Tanshionones are a class of diterpenes derived from the dried roots and rhizomes of *Salvia miltiorrhiza* (Danshen). Tanshinone I (TI) is currently the only tanshinone found to significantly inhibit the growth of triple-negative breast cancer (TNBC) cells (Gong et al., 2012). In breast cancer, TI is shown to significantly enhance paclitaxel chemosensitivity by inhibiting autophagy via AKT/p38 MAPK pathway-mediated blockade of autophagosome-lysosome fusion, while preserving minimal toxicity to normal cells (Tang et al., 2024).

#### 3.3.6 Atractylenolide I

Atractylenolides are sesquiterpenoids derived from *Atractylodes macrocephala.* All atractylenolides exhibit antitumor properties in various types of cancer through a mechanism involving inhibition of tumor cell proliferation (Jiang et al., 2022). Atractylenolides, particularly AT-I, exhibit significant immunomodulatory effects by promoting tumor antigen presentation in both human and mouse CRC cells, thereby enhancing CD8^+^ T cell-mediated cytotoxicity (Xu et al., 2021a). In addition, AT-I has been shown to reverse immune evasion in lung, colorectal, and ovarian cancers (Liu et al., 2016), improving the response to chemotherapy and suppressing metastatic growth by modulating immune cell functions and regulating exosome-mediated signaling (Liu et al., 2020).

### 3.4 Phenolic acids

Phenolic acids, abundant in dietary sources such as fresh fruits and vegetables, exhibit significant anticancer effects by inhibiting tumor cell proliferation and inducing apoptosis. In addition to these direct effects, they modulate immune responses by enhancing T cell activation, inhibiting immune-suppressive cells (such as Tregs and MDSCs), and restoring immune surveillance in the TME. This immune modulation can improve chemotherapy efficacy and potentially overcome chemoresistance. Due to their natural abundance, favorable safety profiles, and multi-targeted mechanisms, phenolic acids are promising adjuvant therapies for enhancing the efficacy and reducing side effects of conventional chemotherapy (Silva et al., 2025; Bakrim et al., 2022).

#### 3.4.1 Chlorogenic acid

Chlorogenic acid (5-O-caffeoylquinic acid, CGA) is a phytochemical produced in dietary plants. CGA exhibits promising anticancer activities through multiple mechanisms, including inhibition of cancer cell cycles, induced apoptosis, and suppressed proliferation (Cortez et al., 2024). CGA sensitizes hepatocellular carcinoma (HCC) cells to 5-fluorouracil (5-FU) treatment by inhibiting ERK activation through ROS overproduction (Yan et al., 2015). In synergy with doxorubicin (DOX) treatment, CGA was shown to significantly reduce cancer cell viability and growth through induced apoptosis by inhibiting ERK activation (Salzillo et al., 2021).

#### 3.4.2 Gallic acid

Gallic acid (GA) is a natural plant-derived triphenol derived from fruits, nuts and green tea. Given its relatively low bioavailability, GA often requires alternative delivery methods, including but not limited to nanocarriers, to enhance its therapeutic efficacy and ensure effective tumor targeting (Xiang et al., 2024). GA and its derivatives exhibit antitumor activity in multiple human cancer cell lines, including lung, breast, prostate, and skin cancer and mitigates cisplatin resistance in ovarian cancer cell lines (Lo et al., 2011; Al Balushi et al., 2022). Wang et al. further discovered that GA administration can induce tumor cell apoptosis and potentiate cisplatin-mediated anticancer effects in the human small cell lung cancer H446 cell line via a ROS-dependent mitochondrial apoptotic pathway (Wang et al., 2016a).

### 3.5 Others

Honokiol, a phenylpropanoid biaryl lignan, inhibits HCC cell proliferation and STAT3 activation by modulating its upstream kinases (c-Src, JAK1, JAK2), leading to upregulation of tyrosine phosphatase SHP-1 and dose-as well as time-dependent tumor cell apoptosis (Rajendran et al., 2012). Through such underlying mechanisms, honokiol potentiates paclitaxel/doxorubicin-induced tumor cell apoptosis, thus presenting as a novel STAT3 blocker that can effective treat HCC and chemoresistant cancers (Rajendran et al., 2012). We summarized the natural products described in this chapter and organized them into Table 1.

**TABLE 1:** Overview of natrual products: effectiveness and mechanisms against various tumor types.

Structural type	Natural product(s)	Structural formula	Tumor type	*In vivo/vitro*	Resistant chemotherapy drug	Pharmacological mechanism	References
Alkaloid	Berberine	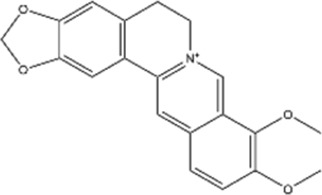	Gastric cancer	*In vitro*	Cisplatin	Sensitization of gastric cancer cells to cisplatin	Kou et al. (2020)
	Camptothecin	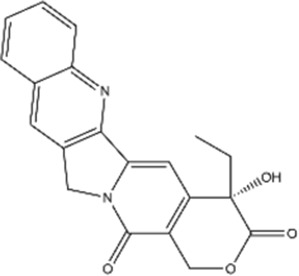	Colorectal cancer	*In vivo/vitro*	CPT-11	Inhibition of cancer progression in combination with CPT-11 and aloin through miRNA-133b activation	Jassi et al. (2023)
	Matrine	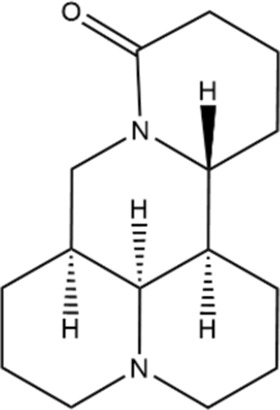	Hepatocelluar carcinoma	*In vivo/vitro*	Cisplatin	Significant anti-metastatic effect on HCC via enhanced miR-199a-5p expression and subsequently impaired HIF-1α signaling and EMT.	Dai et al. (2021)
	Ligustrazine	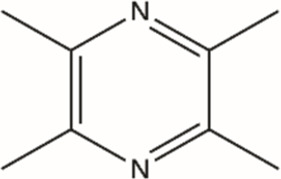	Drug-resistant hepatocellular carcinoma	*In vivo/vitro*	5-FU	Antitumor effects in liver cancer by increased ROS levelspecies, TrxR inhibition, reduced mitochondrial membrane potential, DNA damage, and modulation of autophagy-related proteins	Qian et al. (2021)
Flavonoids	Baicalin	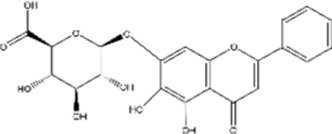	Hepatocelluar carcinoma	*In vitro*	5-FU	Reversed drug resistance in Bel-7402/5-FU cells by inhibiting the PI3K/AKT pathway, promoted autophagy and apoptosis to restore chemosensitivity	Li et al. (2025a)
	Baicalein	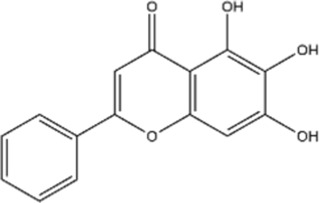	Cervical cancer	*In vitro*	Cisplatin	Potential enhancement of the sensitivity to cisplatin through the Akt signaling pathway, ultimately inducing cuproptosis	Jin et al. (2024)
	Resveratrol	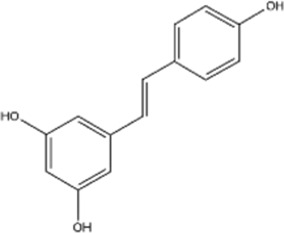	Lung cancer	*In vivo/vitro*	Osimertinib	Mitigates osimertinib resistance by targeting BCL2L11 to promote apoptosis	Yu et al. (2025)
	Quercetin	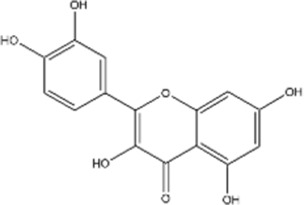	Human cervical cancer	*In vitro*	Cisplatin	Enhanced cisplatin chemosensitivity through induced tumor cell apoptosis, inhibited proliferation, migration and invasion	Xu et al. (2021b)
	Curcumin	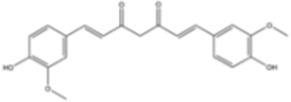	Colorectal cancer	*In vitro*	Oxaliplatin	Inhibited EMT via regulation of TGF-β/Smad2/3 signaling pathway	Yin et al. (2019)
	EGCG	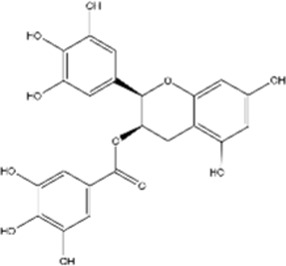	Cisplatin-resistant oral cancer	*In vitro*	Cisplatin	Inhibition of cancer progression by MDR1 downregulation, reduced phosphor-AKT (Ser473)/STAT3 signaling, and reduced Bcl-2 levels	Yuan et al. (2017)
	Naringin	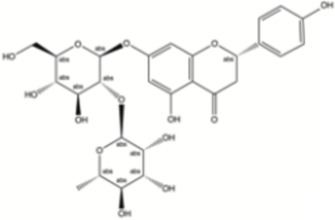	Cervical cancer	*In vitro*	Cisplatin	Induced RIP3/MLKL-mediated necroptosis, inhibited cell cycle progression and cell invasion-related proteins, and potentiated the anti-tumor effect of cisplatin	Martínez-Rodríguez et al. (2021)
Terpenoids	Ginsenoside Rg3	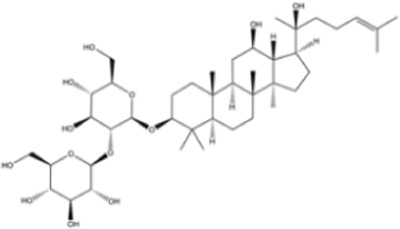	Lung cancer	*In vitro*	Cisplatin	Suppression of PD-L1 expression and enhanced T cell function	Jiang et al. (2017)
	Ginsenoside RH2	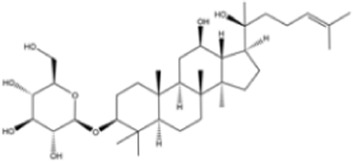	Lung adenocarcinoma	*In vitro*	Cisplatin	Repressed EGFR/PI3K-AKT signaling, autophagy, and cisplatin-induced PD-L1 expression	Chen et al. (2020b)
	Cycloastragenol	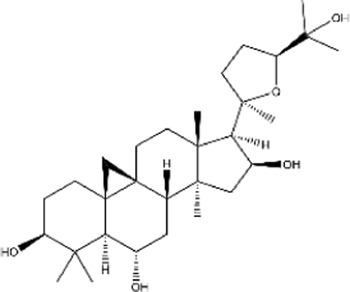	Colorectal cancer	*In vivo*	PD-1 antibodies	Enhanced CD8^+^ T cell cytotoxicity enhanced tumor killing effects in combination with PD-1 antibody	Deng et al. (2022)
	Triptolide	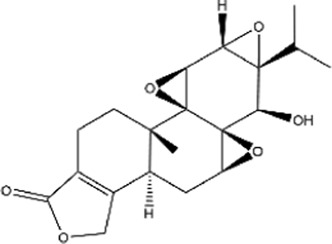	Ovarian carcinoma	*In vitro*	Cisplatin	Enhanced cisplatin sensitivity in platinum-resistant cell line	Huang et al. (2019)
	Demethylzeylasteral	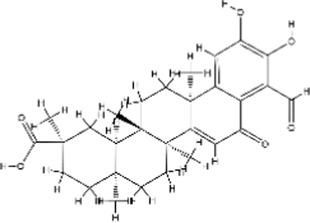	Colorectal cancer	*In vivo*	PD-1 antibodies	Enhanced T-cell mediated tumor cytotoxicity via downregulated PD-L1 expression	Zhang et al. (2024b)
	Tanshinone I	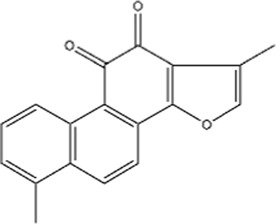	Triple-negative breast cancer (TNBC)	*In vivo/vitro*	Paclitaxel	Enhanced paclitaxel chemosensitivity by inhibiting autophagy via the AKT/p38 MAPK signaling pathway	Tang et al. (2024)
Phenolic acids	Chlorogenic acid	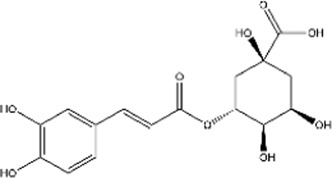	Hepatocellular carcinoma	*In vitro*	5-FU	Enhanced chemosensitivity to 5-fluorouracil (5-FU) treatment by inhibiting ERK activation through ROS overproduction	Yan et al. (2015)
	Gallic acid	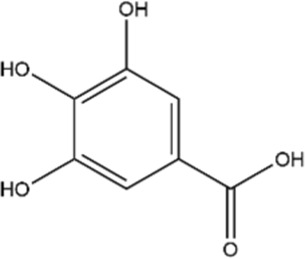	Human small cell lung cancer	*In vitro*	cisplatin	Induced tumor cell apoptosis; enhanced cisplatin-mediated anticancer effects via ROS-dependent mitochondrial apoptotic pathway	Wang et al. (2016a)
Uncategorized	Honokiol	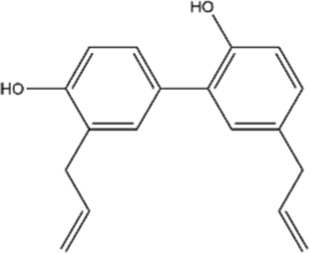	Hepatocellular carcinoma	*In vitro*	Paclitaxel/doxorubic	Inhibition of cell proliferation via modulated STAT3 activation; Enhanced chemosensitivity to paclitaxel/doxorubic treatment via induced apoptosis	Rajendran et al. (2012)

## 4 Mechanisms through which natural products influence immunity to alleviate or overcome tumor drug resistance

### 4.1 Modulation of immune cell functions

Current studies have identified key strategies for improving tumor drug resistance, including (i) reshaping the immune microenvironment; (ii) targeting of immunosuppressive cells (such as eliminating Tregs or blocking the functions of MDSCs); and (iii) activation of effector immune cells (such as enhancing the infiltration of CD8^+^ T cells).

#### 4.1.1 Enhancement of T-cell activity

In the TME, T cell activity is crucial for immune responses that eliminate tumor cells. Small molecule natural products have shown significant potential for promoting T cell proliferation, alleviating T cell exhaustion, and restoring their cytotoxic function against tumor cells. Evidence suggests that natural products can function to assist T cells in overcoming tumor chemoresistance through the remodeling of the immunosuppressive microenvironment. For example, EGCG actively restores effector function in exhausted CD8^+^ T cells via PD-L1 suppression and granzyme B induction. This approach offers a novel strategy to augment anti-tumor immunity in melanoma (Ravindran Menon et al., 2021). Moreover, 4′-hydroxyphenyl-deficient EGCG metabolites selectively enhance CD4^+^ T cell activation by promoting IFN-γ secretion and STAT1 phosphorylation, thereby augmenting adaptive immune responses against tumor cells (Kim et al., 2016). As another example, curcumin exhibits promising therapeutic effects in head and neck squamous cell carcinoma (HNSCC). It acts to alleviate CD8^+^ T cell exhaustion in the TME by enhancing T-cell proliferation, increasing tumor-infiltrating lymphocytes (TILs), and downregulating immunosuppressive checkpoint molecules (e.g., PD-1, TIM-3, PD-L1, PD-L2, Galectin-9), thereby restoring anti-tumor cytotoxicity (Lihua et al., 2021).

Therapies that employ specific antibodies, such as programmed cell death-1 (PD-1)/programmed cell death ligand-1 (PD-L1) blocking therapy, have been widely used in cancer immunotherapy, Nevertheless, it is essential to develop therapies that utilize small-molecule checkpoint inhibitors with more favorable pharmacokinetics (Yang et al., 2020).

Berberine (BBR) inhibits CSN5 deubiquitination activity, leading to PD-L1 degradation and enhanced tumor-infiltrating T-cell activation in NSCLC. This offers a novel immune checkpoint modulation strategy and a promising therapeutic approach for NSCLC (Yang et al., 2020). *In vivo* evidences suggest that demethylzeylasteral exerts antitumor effects by binding to the deubiquitinating enzyme USP22 to promote its proteasomal degradation, which subsequently enhances PD-L1 ubiquitination and degradation, ultimately potentiating T cell-mediated cytotoxicity against cancer cells and suppressing tumor growth (Zhang et al., 2024b). Rg3 overcomes cisplatin resistance in NSCLC by dual targeting of PD-L1 immune checkpoint (via Akt/NF-κB inhibition) and T cell functional restoration (Jiang et al., 2017). Rosmarinic acid combined with ginsenoside Rg1 co-inhibits the COX-2 and PD1/PD-L1 signaling axes, leading to suppression of colon cancer metastasis and improvements to immune responses at different concentration levels (Liu et al., 2024).

#### 4.1.2 Regulation of macrophage polarization

TAMs, derived from peripheral blood monocytes recruited to the TME via chemotactic signals (e.g., CCL2/CCR2 axis), polarize into two distinct phenotypes, M1 and M2 (see Section 2.2.2 for details). Berberine exhibits anti-tumor effects through TME reprogramming by inhibiting IL-6/STAT3 signaling to suppress M2 macrophage polarization and pro-inflammatory cytokine secretion (e.g., IL-10, TGF-β), while concurrently promoting M1 macrophage activation and CD4^+^/CD8^+^ T cell cytotoxicity via upregulation of MHC-II/CD40 and IFNγ production (Dhruvi et al., 2022). Baicalin promotes M2 macrophage polarization and angiogenesis by upregulating VEGF/VEGFR expression, which in turn induces PD-L1 expression via autocrine VEGF signaling in M2 macrophages, leading to a dampening of T cell-mediated anti-tumor immunity. This mechanism could potentially alleviate drug resistance in cancer treatments (Lai et al., 2019). Baicalein also shows anti-breast cancer effects by modulating M2 macrophage polarization and reducing TGF-β secretion (Zhao et al., 2018).

#### 4.1.3 Regulation of natural killer (NK) cell function

NK cells are critical innate immune effectors capable of eliminating tumor cells without prior sensitization. Recent studies highlight that natural products can enhance NK cell cytotoxicity and tumor infiltration, thereby overcoming drug resistance.

EGC-M5, a major EGCG metabolite, is shown to potently induce NK cell cytotoxicity through perforin/granzyme B-mediated tumor cell lysis and downregulation of PD-L1/B7-H3 immune checkpoints. It provides a novel strategy to utilize innate immunity in cancer therapy (Kim et al., 2016). Oridonin enhances NK cell-mediated immunosurveillance against lung cancer by orchestrating functional reprogramming of NK cells (e.g., degranulation, cytokine production) and disrupting tumor immune evasion via STAT3 inhibition (Hwang and Chang, 2023). Afolabi et al. demonstrated that PL exhibits *in vitro* antitumor/proliferative activity and enhances NK cell-mediated cytolysis of tumor cells without impairing NK function. This is achieved by inducing misfolded proteins, blocking autophagy, overproducing ROS, and facilitating activation receptor-ligand interactions, while suppressing both MHC-I-deficient and -sufficient tumor growth *in vivo*. Such evidence indicates that natural compounds are immunomodulatory and can influence NK cell behaviors to influence tumor cell elimination and growth (Afolabi et al., 2021).

#### 4.1.4 Regulation of dendritic cell function

DCs, the most potent specialized antigen-presenting cells (APCs) in the TME, play pivotal roles in tumor immunity. Upon maturation and activation, DCs present cancer-associated antigens and upregulate MHC molecules and costimulatory ligands, thereby initiating the adaptive immune response of naive T lymphocytes. Given their crucial role in orchestrating immune responses within the TME, DC-based vaccines have emerged as a promising immunotherapeutic strategy for activating CTLs to combat tumors (Zhang et al., 2025b). Nevertheless, how to further enhance the functionality of DCs in antitumor therapies remains a key area of research (Gardner et al., 2020; Perez and De Palma, 2019). Evidence indicates that EGCG promotes DC maturation and, when combined with mesothelin - specific DNA vaccine, enhances anti-cancer effects, which offers a potential immunotherapy opportunity for mesothelin - expressing cancers (Chen et al., 2018). Plantain polysaccharide (PLP) upregulates DC maturation both *in vitro* and *in vivo*. PLP-activated DCs stimulate lymphocyte proliferation and differentiate naive T cells into cytotoxic T cells (Gao et al., 2021).

#### 4.1.5 Regulation of myeloid-derived suppressor cells

Current evidence indicates that MDSCs suppress CTLs via upregulation of ARG1, iNOS, and ROS (Bronte and Zanovello, 2005; Wu et al., 2022). For example, overexpression of *ARG1* in MDSCs depletes L-arginine in the TME, hindering CTL activation and T cell proliferation (Grzywa et al., 2020). In a murine breast cancer model, EGCG suppresses NF-κB in MDSCs in a dose-dependent manner, reducing downstream immunosuppressive mediators like ARG1, iNOS, NO, IL-6, IL-10, and TGF-β. EGCG also inhibits MDSCs growth while promoting apoptosis *in vitro*, mitigating MDSC-mediated immunosuppression (Xu et al., 2020).

### 4.2 Regulation of immune-related signaling pathways

#### 4.2.1 NF–κB signaling pathway

NF-κB, a transcription factor activated via ubiquitination-dependent degradation of IκB in response to inflammatory stimuli or chemotherapeutic stress, can translocate to the nucleus to upregulate genes involved in cell survival (e.g., Bcl-2), inflammation (e.g., TNF-α, IL-6), and multidrug resistance (e.g., P-gp), thereby facilitating tumor progression and chemoresistance (Meng et al., 2020; Tan et al., 2019; Lee et al., 2019). Naringin reverses cisplatin resistance in ovarian cancer by inhibiting NF-κB and its downstream target COX-2, providing a mechanistic basis for its potential therapeutic role against platin-resistant tumors (Zhu et al., 2017).

#### 4.2.2 JAK-STAT signaling pathway

Constitutively activated STAT3 suppresses tumor proinflammatory mediator expression and inhibits DC maturation, forming a tumor-to-DC immune evasion cascade (Wang et al., 2023). BBR-mediated JAK1-STAT3 inhibition via miR-17-5p upregulation induces apoptosis in p53-deficient BCa cells and senescence in p53-proficient cells (Yangyang et al., 2021). BBR exerts anti-gastric cancer effects *in vitro* and *in vivo* by inducing apoptosis, G0/G1 cell cycle arrest, and inhibiting migration/invasion via suppression of the IL-6/JAK2/STAT3 signaling axis, thereby representing a multi-targeted therapeutic candidate for overcoming chemoresistance in gastric carcinoma (Xu et al., 2022). Future investigations should consider targeting this oncogenic pathway to enhance proinflammatory signals that activate innate immunity and DCs, driving tumor-specific T-cell responses while modulating the IL-6/STAT3/PD-1 axis in CD8^+^ PD-1^+^ T cells within the TME to inhibit drug resistance.

#### 4.2.3 MAPK signaling pathway

Signaling through the MAPK signaling pathway enables tumor cells to evade the cytotoxic effects of platinum chemotherapy by promoting cell survival, inhibiting apoptosis, and regulating cellular stress responses (Bahar et al., 2023; Haagenson and Wu, 2010). Tanshinone I enhances paclitaxel chemosensitivity in breast cancer by targeting the AKT/p38 MAPK pathway to inhibit autophagy, specifically blocking autophagosome-lysosome fusion without affecting lysosomal acidification (Tang et al., 2024).

#### 4.2.4 PI3K - Akt–mTOR signaling pathway

Constitutive activation of the PI3K/AKT axis contributes to cisplatin resistance in SKOV3/DDP ovarian cancer cells. Moreover, combinatorial treatment with triptolide (TPL) and cisplatin (DDP) significantly attenuates tumor growth *in vivo* by downregulating p-PI3K/p-AKT/Survivin signaling and upregulating caspase-3-mediated apoptosis, generating a stronger synergistic effect compared to monotherapy (Huang et al., 2019). Berberine enhances cisplatin sensitivity in cancer cells by downregulating drug transporters MDR-1/MRP1, promoting apoptosis via caspase activation, and inhibiting PI3K/AKT/mTOR signaling, thereby overcoming chemoresistance through dual targeting of drug efflux and prosurvival pathways (Kou et al., 2020). Baicalein induce cuproptosis through the Akt pathway, which shows its potential to improve the sensitivity of cervical cancer cells to cisplatin (Jin et al., 2024). In cisplatin-resistant CAR cells, EGCG treatment leads to dose-dependent suppression of MDR1 and anti-apoptotic proteins Bcl-2, p-AKT (Ser473), and p-STAT3, indicating its potential for chemosensitization (Yuan et al., 2017).

#### 4.2.5 AMPK signaling pathway

**FIGURE 2:**
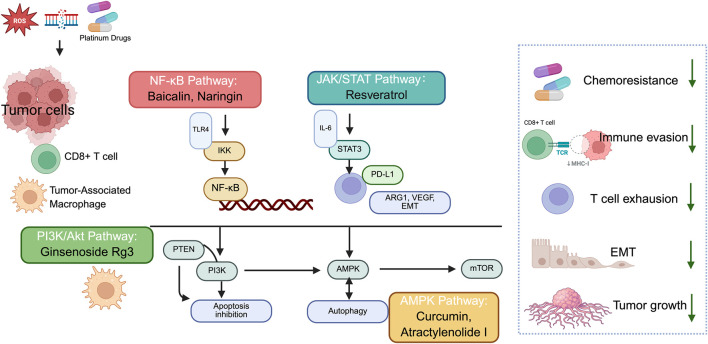
Summary of the immunomodulatory mechanisms by which natural products alleviate and overcome platinum-based chemoresistance within the TME. Platinum-based chemotherapy and tumor microenvironmental stimuli activate NF-κB, JAK/STAT, PI3K-Akt, and AMPK signaling pathways in tumor cells and immune cells. Aberrant activation of NF-κB and JAK/STAT signaling promotes immune evasion through upregulation of PD-L1, inflammatory cytokines, and immunosuppressive mediators, while PI3K-Akt signaling enhances tumor cell survival, metabolic reprogramming, and resistance to apoptosis. AMPK signaling acts as a context-dependent metabolic regulator that modulates autophagy and stress adaptation under chemotherapeutic pressure. Natural products such as baicalin, naringin, ginsenosides, and atractylenolide I target these pathways to restore antitumor immunity, enhance CD8^+^ T cell and NK cell-mediated cytotoxicity, and sensitize tumors to platinum-based chemotherapy.

The AMP-activated protein kinase (AMPK) signaling pathway affects cancer chemoresistance by metabolic reprogramming and regulating stress responses, including autophagy and survival pathways that enable tumor cells to withstand chemotherapeutic stress and restore proliferative capacity (Wang et al., 2016b; McNamee et al., 2024). Berberine overcomes doxorubicin resistance in breast cancer cells via dose-dependent AMPK signaling. It sensitizes cells through AMPK-HIF-1α-P-gp axis at low doses, and directly induces apoptosis via AMPK-p53 pathway independently of HIF-1α high doses, as reported through *in vitro* and *in vivo* studies (Pan et al., 2017) The summary of the above signaling pathways is presented in Figure 2.

### 4.3 Induction of immunogenic cell death in tumor cells

Immunogenic cell death (ICD), a specialized apoptotic pathway, enables cancer cells to act as “therapeutic vaccines” by inducing antitumor immunity without adjuvants. The cellular mechanism is considered a key mechanism in cancer-specific immunotherapies such as preventive and therapeutic cancer vaccines (Son et al., 2016).

Preclinical evidence demonstrates that RES exhibits antitumor activity in ovarian cancer by inducing ICD and modulating cytokine profiles, which is characterized by suppressed TGF-β secretion and enhanced production of pro-inflammatory cytokines IL-12 and IFN-γ (Zhang et al., 2019). Rg3, induces ICD in both immunogenic (B16F10 melanoma) and non-immunogenic (Lewis lung carcinoma, LLC) tumor cells by promoting apoptosis, upregulating surface markers of ICD (calreticulin, heat shock proteins), and enhancing IFN-γ secretion while suppressing pro-angiogenic (TNF-α) and immunosuppressive (TGF-β) cytokines. This effect promotes DC uptake of dying tumor cells as evidenced by increased CRT^+^ CD11c^+^ cells, suggesting the potential for Rg3 to be used as an immunotherapeutic treatment that integrates antitumor cytotoxicity with DC-mediated immune activation (Son et al., 2016).

## 5 Conclusion and future directions

We summarized the therapeutic potential of diverse natural products—including alkaloids, flavonoids, terpenoids, phenolic acids, and other structural classes—in reversing tumor drug resistance, with a focus on platinum-based chemotherapies and targeted therapies. Tumor resistant to chemotherapeutics agents (cisplatin, oxaliplatin, 5-fluorouracil, paclitaxel) and targeted agents (sorafenib, osimertinib, gefitinib) can be effectively sensitized by natural products through multiple mechanisms. For instance, alkaloids such as berberine and matrine modulate drug transporters (MDR1, MRP1) and signaling pathways (PI3K/AKT, NF-κB); flavonoids, including baicalin, curcumin, and naringenin, regulate immune cell functions (CD8^+^ T cells, macrophages, NK cells) and induce necroptosis or ferroptosis; terpenoids like ginsenosides and atractylenolide I enhance tumor antigen presentation and T cell-mediated cytotoxicity; and phenolic acids, including chlorogenic acid and gallic acid, overcome resistance via ROS-mediated pathways. Collectively, these natural products act as multitarget immunomodulators and signaling regulators, reshaping the immunosuppressive tumor microenvironment, inhibiting oncogenic pathways, and restoring chemosensitivity.

Despite the promising preclinical results, the translation of these findings into clinical practice remains challenging. Many natural products exhibit limited pharmacokinetic properties, including low bioavailability, rapid systemic clearance, and lack of tumor-specific targeting. Furthermore, existing animal models often fail to recapitulate the complexity of the human TME and thus constrains the predicitive value of preclinical studies for human therapy. Bridging the gaps between preclinical models and clinical application is essential to acclerate the development of natural products-based treatments for chemoresistant cancer (Zhang et al., 2022). In this context, the global diversity of ethnopharmacological knowledge provides a rich resource for identifying novel natural compounds and therapeutic applications, while nanotechnology-based delivery systems offer innovative solutions to overcome pharmacokinetic limitation. Together, these two approches warrant in-depth exploration to fully realize the potential of natural products in cancer therapy.

### 5.1 Current limitations

#### 5.1.1 Lack of clinical trials

Despite promising preclinical evidence, clinical translation of natural products remains severly limited. For example, a high-bioavailability formulation of curcumin (BCM-95) has been tested for its anti-inflammatory and anticancer properties; however, phase II/III clinical trials in cancer patients are still lacking, underscoring challenges in dose optimization and patient selection for natural product-based therapies (ClinicalTrials.gov ID: NCT07248020). Furthermore, systematic clinical trials evaluating the immunomodulatory and anti-cancer effects of natural products are scarce, despite significant efficacy observed *in vitro* and in animal models especially for solid tumors (Hong and Lyu, 2025b). This disconnect between abundant preclinical findings and the paucity of controlled clinical studies highlights the urgent need for comprehensive clinical trials to bridge this gap (Wang et al., 2023).

#### 5.1.2 Inadequate modeling of TME

The TME consists of complex, heterogeneous populations of immune and stromal cells, which poses significant challenges in extrapolating the preclinical findings in the effect of natural product to human patients. Conventional 2D cell culture models fail to replicate the intricate cellular interactions of the TME. Although 3D organoid models more accurately reflect human TME dynamics, their use in studying natural product immunomodulation remains limited (Hong and Lyu, 2025b). Immunocompetent animal models have been shown to better replicate TME complexity, but the lack of established protocols for incorporating immune checkpoint inhibition in such models restricts their utility for assessing natural products’ immunomodulatory effect. These limitations in TME modeling impede our understanding of how natural products interact with immune responses in human cancers (Shah and D’Souza, 2025; Crouigneau et al., 2024).

#### 5.1.3 Mechanistic gaps

While natural products exhibit potent anticancer and immunomodulatory effects in preclinical studies, their underlying molecular mechanisms remain insufficiently understood. Key gaps exist in elucidating the interaction between direct cytotoxicity and immune activation in human cancers. For example, flavonoids and terpenoids have been shown to modulate macrophage polarization and T-cell functions, but the specific signaling pathways and molecular interactions involved remain unclear (pmc.ncbi.nlm.nih.gov). Moreover, preclinical studies often employ doses far exceeding those achievable in patients, raising concerns about the clinical relevance of observed mechanisms (Hong and Lyu, 2025a) (mdpi.com). These issues highlight the need for more rigorous mechanistic studies using human-relevant models to establish clinically translatable pathways for natural product-based therapies (Chunarkar-Patil et al., 2024).

#### 5.1.4 Pharmacokinetic and targeting barriers

Despite the promising anticancer activities in preclinical models, many natural products face substantial pharmacokinetic and targeting limitations that hinder clinical translation. One of the most pervasive challenges is poor oral bioavailability, which severely limits systemic exposure and therapeutic efficacy. For instance, curcumin demonstrates robust anti-inflammatory and anticancer effects *in vitro*, but its clinical application has been hampered by rapid metabolism, poor absorption, and short half-life, resulting in low plasma levels and inconsistent treatment outcomes in cancer patients unless bioavailability-enhancing formulations are used (e.g., nanoparticulate or phospholipid complexes) to improve pharmacokinetics and tissue distribution (Kunnumakkara et al., 2023).

Similarly, other natural compounds, such as hydrophobic phytochemicals polyphenols and flavonoids, exhibit limited solubility and rapid metabolic clearance, which leads to insufficient target engagement and suboptimal therapeutic concentrations in tumor tissues (Naeem et al., 2022). Non-specific tissue distribution further diminishes efficacy and increases the potential for off-target effects. Nanoparticle-based encapsulation of poorly soluble compounds, such as plant sterols or alkaloids, has shown promise in enhancing both bioavailability and tumor-specific uptake in preclinical studies, yet these strategies have rarely advanced to clinical testing (Andreani et al., 2024).

Moreover, differences in metabolism between humans and animal models exacerbate the translational gap: compounds that are active *in vitro* or in rodent models fail to achieve comparable plasma exposure or tumor penetration in humans. Collectively, these obstacles—low bioavailability, rapid systemic clearance, and inadequate targeting—underscore the need for advanced drug delivery systems, semisynthetic analog optimization, and rigorous clinical pharmacokinetic studies to enable reliable clinical efficacy of natural products (Goel and Jain, 2024).

#### 5.1.5 Underexploration of ethnopharmacological diversity

A critical yet underexplored limitation is the underutilization of global ethnopharmacological knowledge in natural product drug discovery for cancer chemoresistance (Iqbal et al., 2025). Traditional medicinal systems worldwide—including Traditional Chinese Medicine (TCM), Ayurveda, African traditional medicine, and Indigenous healing practices—have accumulated centuries of empirical knowledge on plant-based remedies for treating diseases with cancer-like symptoms. However, many of these traditional formulations and their bioactive constituents remain uncharacterized in the context of modern cancer immunotherapy and chemoresistance reversal. This gap limits the discovery of novel lead compounds and overlooks the cultural and ecological insights that could inform optimal extraction, formulation, and therapeutic application (Zou et al., 2024).

### 5.2 Future strategies

#### 5.2.1 Development of 3D organ-on-a-chip models

To bridge the gap between preclinical findings and clinical translation, the development of advanced 3D organoid-immune co-culture systems is crucial. These systems could more accurately mimic the human TME and provide a platform for evaluating the immunomodulatory effects of natural products before progressing to clinical trials. By improving the predictability of preclinical studies, these models can help optimize drug delivery, dosing, and immune modulation strategies (Wang et al., 2025; Noorintan et al., 2024).

#### 5.2.2 Optimization of combination therapies

Combining natural products with ICIs, targeted therapies, or chemotherapy offers a promising range of options to approach the challenge of overcoming drug resistance in cancer. However, the mechanisms of synergy or antagonism between these therapies need to be carefully explored. Studies should focus on how natural molecules modulate the TME to enhance chemosensitivity or reverse T cell exhaustion to potentiate the effects of ICIs. Furthermore, the optimal drug sequencing, dose scheduling, and patient stratification based on tumor immune phenotypes are critical to maximizing the therapeutic efficacy of combination treatments (Zhou et al., 2025a).

#### 5.2.3 Compound structural optimization and nanodelivery

While many natural products have demonstrated therapeutic potential, their pharmacokinetics and bioavailability present as barriers that limit clinical success. Future efforts should focus on structural optimization of these compounds to enhance their tumor-targeting and bioavailability. The development of nanoparticle-based drug delivery systems or liposomal formulations could improve the targeting efficiency and sustained release of these compounds, thus improving their clinical applicability (Huang et al., 2024). Key nanodelivery platforms with well-characterized mechanisms include biodegradable polymeric nanoparticles (PEG, PLGA, chitosan) (Yilmaz et al., 2026), cell membrane-mimetic liposomes(Uddin et al., 2026), high-loading mesoporous silica nanoparticles (MSNs) (Xie et al., 2023), and biocompatible extracellular vesicles (EVs) (Xu et al., 2025). These systems encapsulate both hydrophilic and hydrophobic natural products, while functionalization with targeting ligands (folic acid, anti-PD-L1/CD44 antibodies) or TME-homing receptors (CXCR4) facilitates active targeting. Moreover, stimuli-responsive designs enable on-demand cargo release in the acidic/oxidative TME, promoting therapeutic efficacy and precision.

#### 5.2.4 Evidence-based medicine and clinical trial design

Clinical trials should be designed with a focus on personalized medicine, incorporating patient immune profiles and genetic backgrounds to guide therapeutic choices. Trials must also ensure appropriate dosing, optimal drug combinations, and the inclusion of immune biomarkers to evaluate both tumor response and immune activation. A more evidence-based approach will help to establish the safety and efficacy of natural products in clinical settings, paving the way for their integration into routine cancer treatment regimens (Momoi et al., 2025).

### 5.3 Concluding remarks

Despite the challenges, the adoption and use of natural products in cancer immunotherapy holds immense potential as therapeutic strategies to overcome treatment resistance and improving clinical outcomes. These compounds offer beneficial impact through distinct cellular and molecular mechanisms, particularly through modulating immune responses and enhancing chemosensitivity. However, to fully realize their clinical potential, more preclinical studies with advanced models, structural optimization, and well-designed clinical trials are required. By addressing these challenges and optimizing the use of natural products in combination with conventional therapies, we can revolutionize cancer treatment with novel, effective therapies that improve patient survival and quality of life.
